# DNA methylation holds prognostic information in relapsed precursor B-cell acute lymphoblastic leukemia

**DOI:** 10.1186/s13148-018-0466-3

**Published:** 2018-03-05

**Authors:** Magnus Borssén, Jessica Nordlund, Zahra Haider, Mattias Landfors, Pär Larsson, Jukka Kanerva, Kjeld Schmiegelow, Trond Flaegstad, Ólafur Gísli Jónsson, Britt-Marie Frost, Josefine Palle, Erik Forestier, Mats Heyman, Magnus Hultdin, Gudmar Lönnerholm, Sofie Degerman

**Affiliations:** 1 Department of Medical Biosciences, Umeå University, Blg 6M, 2nd floor, SE-90185 Umeå, Sweden; 2 Department of Medical Sciences and Science for Life Laboratory, Uppsala University, Uppsala, Sweden; 3 Children’s Hospital, Helsinki University Central Hospital, University of Helsinki, Helsinki, Finland; 4 Department of Paediatrics and Adolescent Medicine, Rigshospitalet, and Institute of Clinical Medicine, University of Copenhagen, Copenhagen, Denmark; 5 Department of Pediatrics, University of Tromsø and University Hospital of North Norway, Tromsø, Norway; 6 Pediatric Hematology-Oncology, Children’s Hospital, Landspitali University Hospital, Reykjavik, Iceland; 7 Department of Women’s and Children’s Health, Pediatrics, University of Uppsala, Uppsala, Sweden; 8 Childhood Cancer Research Unit, Department of Women’s and Children’s Health, Karolinska Institutet, Karolinska University Hospital, Stockholm, Sweden

**Keywords:** DNA methylation, BCP-ALL, Prognosis, CIMP, Relapse, Risk stratification

## Abstract

**Background:**

Few biological markers are associated with survival after relapse of B-cell precursor acute lymphoblastic leukemia (BCP-ALL). In pediatric T-cell ALL, we have identified promoter-associated methylation alterations that correlate with prognosis. Here, the prognostic relevance of CpG island methylation phenotype (CIMP) classification was investigated in pediatric BCP-ALL patients.

**Methods:**

Six hundred and one BCP-ALL samples from Nordic pediatric patients (age 1–18) were CIMP classified at initial diagnosis and analyzed in relation to clinical data.

**Results:**

Among the 137 patients that later relapsed, patients with a CIMP− profile (*n* = 42) at initial diagnosis had an inferior overall survival (pOS_5years_ 33%) compared to CIMP+ patients (*n* = 95, pOS_5years_ 65%) (*p* = 0.001), which remained significant in a Cox proportional hazards model including previously defined risk factors.

**Conclusion:**

CIMP classification is a strong candidate for improved risk stratification of relapsed BCP-ALL.

**Electronic supplementary material:**

The online version of this article (10.1186/s13148-018-0466-3) contains supplementary material, which is available to authorized users.

## Introduction

Using modern contemporary chemotherapy protocols for childhood acute lymphoblastic leukemia (ALL) with treatment stratification based on cytogenetic features, immunophenotype and early response through quantification of minimal residual disease, the 5-year overall survival now exceeds 90% [1]. Despite improved results of primary treatment, relapse is still the most common consequence from treatment failure, and less progress has been achieved for therapy outcome after relapse with an overall survival rate of ≈ 55% in the Nordic countries [2]. Few well-defined risk factors apart from time to relapse exist at relapse, which impedes risk stratification after relapse.

DNA methylation is a central epigenetic mechanism and an increasing body of evidence highlights the importance of epigenetics in cancer biology [3]. Pediatric ALL has been extensively characterized from a DNA methylation perspective, and the methylation pattern has been shown to reflect both cytogenetic aberrations and immunophenotype [4]. We have previously shown prognostic relevance of promoter associated DNA methylation in T-cell acute lymphoblastic leukemia (T-ALL), where patients displaying a less methylated CpG island methylator phenotype (CIMP−) profile, based on a defined set of CpG sites, were associated with worse prognosis than patients exhibiting a more methylated CIMP+ profile [5]. This finding was subsequently validated in a recent Nordic cohort of high-risk T-ALL patients (MRD > 0.1% at day 29) [6].

Several studies have attempted to ascertain prognostic relevance from DNA methylation status at diagnosis from B-cell precursor acute lymphoblastic leukemia (BCP-ALL) patients [7, 8]. The overlap of prognostically relevant signatures in BCP-ALL thus far is low, probably due to the influence on cytogenetic alterations on epigenetic signatures [8]. However, a general finding from these studies is that when patients are dichotomized based on DNA methylation status, a worse outcome is observed in the group with lower DNA methylation levels [7, 8].

In the present study, we aimed to specifically investigate if the T-ALL trained CIMP profile also holds prognostic relevance in BCP-ALL, potentially giving a broader clinical relevance of the CIMP profile in acute lymphoblastic leukemia in general. We used genome-wide DNA methylation data from a large, well-characterized cohort of BCP-ALL patients, to evaluate the prognostic relevance of CIMP classification at diagnosis and relapse.

## Materials and methods

This study included 601 well-characterized diagnostic and 23 relapse pediatric BCP-ALL samples from children (1–18 years) diagnosed in the Nordic countries (1996–2008) and treated according to the NOPHO ALL 1992 and 2000 protocols [1]. DNA methylation data from previously published Illumina HumanMethylation450K arrays (Gene Expression Omnibus repository accession number GSE49031) [8] were used to classify the BCP-ALL samples as CIMP+ or CIMP− based on percentage of methylated CpG sites within the 1293 CpG site CIMP panel, originally defined in T-ALL datasets [5, 6]. Approximately, 2/3 of the CpG sites in the CIMP panel were relevant for BCP-ALL, and therefore the percentage of methylated CpG sites within the panel separating the CIMP +/− subgroups was adjusted for the BCP-ALL samples to > 25% and ≤ 25%, respectively (Additional file 1: Figure S1). CIMP classification was verified in DNA extracted from 15 patients by a six-gene high-resolution melting (HRM) curve gene panel (Additional file 1: Figure S2). Detailed description of materials and methods are provided in the Additional file 1.

## Results and discussion

**Fig. 1:**
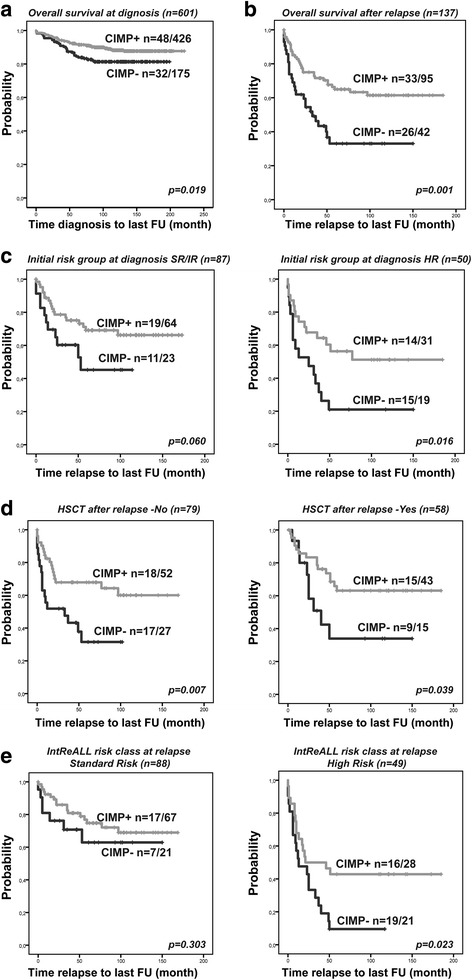
Kaplan-Meier overall survival analysis based on CIMP subgroups in BCP-ALL. Overall survival analysis in **a** 601 diagnostic BCP-ALL samples CIMP classified at diagnosis. Follow-up time (months) from diagnosis to last follow-up. **b**–**e** One hundred and thirty-seven relapsed BCP-ALL samples CIMP classified at diagnosis and stratified for **c** initial risk-group at primary diagnosis (SR/IR and HR), based on age at diagnosis, immunophenotype, cytogenetic aberrations, WBC, and CNS involvement. **d** Hematopoietic stem cell transplantation after relapse (yes/no). **e** IntReALL (International Study for Treatment of Childhood Relapse ALL) risk class (SR/HR) at relapse, based on site of relapse and time from diagnosis to relapse. Follow–up time (months) from relapse to last follow up (**b**–**e**)

The 601 diagnostic pediatric BCP-ALL samples were classified based on their DNA methylation phenotype (*n* = 175 CIMP− and *n* = 426 CIMP+) and analyzed in relation to clinical characteristics. The cytogenetic aberrations were non-randomly distributed among the CIMP subgroups at diagnosis (*p* < 0.001) (Additional file 1: Table S1). The CIMP+ group was enriched for favorable cytogenetic subtypes, i.e., t(12;21)/*ETV6-RUNX1* (88% CIMP+) and HeH (63% CIMP+), whilst the CIMP− group was enriched for patients with unfavorable cytogenetics, i.e., t(1;19)/*TCF3-PBX1* (83% CIMP−) and t(9;22)/*BCR-ABL1* (67% CIMP−) subtypes (Additional file 1: Table S1). The overall survival was significantly lower in the CIMP− group compared with the CIMP+ group (_p_OS_5years_ 85% vs 92%, *p* = 0.019, Fig. 1a), but CIMP status at initial diagnosis of ALL was not associated with relapse (*p* = 0.520) or event-free survival (*p* = 0.424) (Additional file 1: Table S1). The fact that CIMP status did not predict _p_CIR_5years_, but showed differences in _p_OS_5years_ indicates differences in response to relapse treatment between the CIMP groups.

Thus, we further analyzed all the relapsed cases in our cohort separately (*n* = 137), of which 42 were classified as CIMP− and 95 as CIMP+ at initial diagnosis. Patients in the CIMP subgroups did not differ with regard to sex, age, or white blood cell counts at primary diagnosis, but there was a higher frequency of unfavorable cytogenetic subtypes within the CIMP− group (Table 1, *p* = 0.021).

**Table 1:** Clinical characteristics of 137 relapsed pediatric BCP-ALL patients that were CIMP classified at ALL diagnosis

Relapsed BCP-ALL patients	CIMP− *n* = 42	CIMP+ *n* = 95	*p* value
Sex male/female	28/14	51/44	ns
Age at primary diagnosis (months)	48	70	ns
(median, range)	(16–185)	(12–211)	
WBC × 10^9^/l at primary diagnosis	20,7	18,6	ns
(median, range)	(2,2–269)	(1,3–274)	
Cytogenetics at primary diagnosis			0.021
Favorable^a^	18 (43%)	50 (53%)	
Unfavorable^b^	11 (26%)	8 (8%)	
Other^c^	13 (31%)	37 (39%)	
Initial risk group at primary diagnosis^d^			ns
SR/IR	23 (55%)	64 (67%)	
HR	19 (45%)	31 (33%)	
Relapse site^e^			0.077
Bone marrow isolated (iBM)	31 (74%)	50 (53%)	
Combined (iBM and iEM)	6 (14%)	24 (26%)	
Extramedullary (iEM)	5 (12%)	20 (21%)	
Time to relapse (median, months)	29,5	35	ns
(Range)	(5–124)	(1–172)	
Very early (VE)	11 (26%)	12 (13%)	
Early (E)	16 (38%)	38 (40%)	
Late (L)	15 (36%)	45 (47%)	
IntReALL risk class at relapse (relapse site/time to relapse)			0.021
Standard risk (L-iBM, iEM, Comb, E-iEM, Comb)	21 (50%)	67 (71%)	
High risk (VE-iBM, iEM, Comb, E-iBM)	21 (50%)	28 (29%)	
HSCT after relapse			ns
Yes	15 (36%)	43 (45%)	
No	27 (64%)	52 (55%)	
_p_OS_5years_	0.33+/−0.08	0.65+/−0.05	0.001

*ns* not significant, *WBC* white blood cell count, *SR/IR* standard risk/intermediate risk, *HR* high risk, *HSCT* hematopoietic stem cell transplantation

^a^Favorable: t(12;21)(p12;q22), high hyperdiploidy (modal chromosome number ≥ 50)

^b^Unfavorable: t(9;22)(q34;q11), t(1;19), MLL rearrangements (11q23), hypodiploidy (modal chromosome number < 45)

^c^Other: non-stratifying or nonspecific cytogenetic aberrations

^d^Described in ref. [12]

^e^One patient data missing

The prognostic relevance of CIMP status at primary diagnosis was analyzed in relation to overall survival after relapse. The CIMP− subgroup had a significantly worse prognosis with an _p_OS_5years_ of 33% after relapse, as compared with 65% for the CIMP+ subgroup (*p* = 0.001) (Table 1, Fig. 1b). In line with our results, previous studies in this and other cohorts have shown a similar trend in inferior clinical outcomes in BCP-ALL patients with less methylated phenotypes at diagnosis [7–9]. However, to our knowledge, no other study has demonstrated that this difference may be explained by outcome after relapse.

To further investigate the prognostic relevance of CIMP status at primary diagnosis for survival after relapse, we integrated previously identified clinical factors of relevance for relapse outcome. Even though current ALL relapse protocols do not include cytogenetic aberrations in risk stratification, we and others have shown that high risk genetic aberrations at diagnosis have prognostic significance even at relapse [2, 10, 11]. Initial risk group classification at primary diagnosis is based on age at diagnosis, immunophenotype, cytogenetic aberrations, WBC, and CNS involvement (Fig. 1c) [12]. After relapse, patients initially classified and treated as standard risk/intermediate risk (SR/IR) had an _p_OS_5years_ of 63%, in contrast to 42% in the high risk (HR) group (*p* = 0.008). CIMP status could further separate the prognosis in these groups, with worse prognosis for CIMP- patients (*p* = 0.002, log-rank adjusted for initial risk group). The CIMP+/SR/IR patients had an _p_OS_5years_ of 69% after relapse compared to 45% for SR/IR CIMP− patients (*p* = 0.06) (Fig. 1c). This disparity was even more pronounced in the HR group, where patients classified as CIMP+ at diagnosis had an _p_OS_5years_ of 56% after relapse compared with only 21% for HR/CIMP− patients (*p* = 0.016) (Fig. 1c). These results demonstrate that by combining initial risk group and CIMP status at diagnosis, the patients with poorest prognosis after relapse can be further stratified.

Hematopoietic stem cell transplantation (HSCT) treatment might influence the OS, but the outcome depends on initial risk group at primary diagnosis, with improved OS in high-risk patients allocated for HSCT [2]. In our cohort, the proportion of HSCT-treated patients after relapse was similar in both of the CIMP groups (Table 1). However, CIMP- status at diagnosis was associated with poorer outcome regardless if patients were allocated to HSCT or not (*p* = 0.001, log-rank adjusted for HSCT) (Fig. 1d).

Time in first complete remission and anatomic site of relapse are two of the most important prognostic factors in relapsed ALL and represent main stratifying factors for relapse therapy [2, 13]. Expectedly, very early relapse (< 18 months from primary diagnosis) had the worst outcome in our cohort followed by early relapse (≥ 18 months from diagnosis to < 6 months after completion of primary therapy) and late relapse (≥ 6 month after completion of primary therapy) with a _p_OS_5years_ at 26, 46, and 77%, respectively. The median time to relapse did not differ significantly between the CIMP groups, although there was a tendency towards relapse sites. Isolated bone marrow relapse was more common among CIMP− patients: 74% (31/42) vs 53% (50/95) in the CIMP+ group (Table 1), but the difference did not reach statistical significance (*p* = 0.077).

Finally, we assigned relapse risk groups based on a combination of anatomical site of relapse and time to relapse from initial primary diagnosis, as defined by the International Study for Treatment of Childhood Relapse ALL (IntReALL) into standard risk (late relapse and early not isolated bone marrow iBM) or high risk (early iBM and very early relapse) groups [2]. We found significantly higher proportions (50 vs. 29%) of high risk characteristics in the CIMP− patients compared with CIMP+ patients (*p* = 0.021) (Table 1). Importantly, CIMP status at diagnosis could separate the prognosis in these risk groups (*p* = 0.014, log-rank adjusted for combined site/time risk classification), particularly in the high-risk group (*p* = 0.023) (Fig. 1e).

We included CIMP status, age, sex, initial risk group at diagnosis, HSCT status, and IntReALL risk classification in a Cox proportional hazards regression analysis for overall survival analysis after relapse. Although the current IntReALL relapse risk grouping (*p* < 0.001, hazard ratio (HR) high risk 3.79) holds the strongest prognostic information, CIMP status at diagnosis (*p* = 0.036, CIMP− HR 1.81) remained a significant prognostic marker for survival at relapse (Table 2). Notably, when including only IntReALL relapse risk class and CIMP status at diagnosis in the Cox regression analysis, both factors were prognostically relevant for survival after relapse; IntReALL relapse risk class (*p* < 0.001, high risk HR 3.27) and CIMP status (*p* = 0.011, CIMP− HR 1.99) (Additional file 1: Table S3). This further indicates that CIMP status at diagnosis holds important molecular phenotype information of the leukemic cells of relevance for prognosis after relapse.

**Table 2:** Cox’s proportional hazard regression analysis of risk factors for overall survival in relapse patients

Risk factor	N	Reference group	HR
CIMP (−/+)	(42/95)	CIMP+	1.81 (1.04–3.16) (*p = 0.036)
Sex (male/female)	(79/58)	Sex = female	0.95 (0.56–1.60)
Age at primary diagnosis (years)	(137)		1.04 (0.97–1.10)
Initial risk group at primary diagnosis (SR/IR and HR)	(87/50)	Risk group = SR/IR	0.96 (0.53–1.76)
HSCT after relapse (no/yes)	(79/58)	HSCT = no	0.55 (0.32–0.97) (*p = 0.040)
IntReALL risk class (relapse site/time to relapse (SR/HR))	(88/49)	Risk group = SR	3.79 (1.98–7.29) (*p < 0.001)

**Fig. 2:**
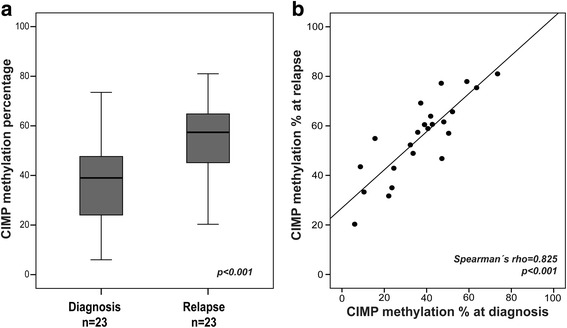
CIMP methylation in paired diagnosis-relapse samples from BCP-patients. **a** CIMP methylation percentage in 23 paired diagnostic and relapse samples from BCP-ALL patients. Mean values at diagnosis and relapse were compared by paired samples *t* test. **b** Scatter plot showing spearman’s rho correlation between CIMP methylation percentage at diagnosis and relapse in 23 BCP-ALL patients

In order to investigate the concordance in CIMP status between diagnosis and relapse samples we compared CIMP status in 23 paired samples from patients with available DNA at both time points. This analysis showed an increased percentage of methylated CpG sites in the CIMP panel at relapse compared to at diagnosis (median 39 vs 57%, *p* < 0.001, Fig. 2a). This was expected since accumulated hypermethylation as a consequence of proliferation has been observed in this material as the relapsed clone(s) have likely undergone additional replication since diagnosis [8]. Importantly, although CIMP methylation increased at relapse, CIMP status at diagnosis was highly correlated CIMP status in the relapsed ALL cells (Spearman’s rho 0.825, *p* < 0.001, Fig. 2b). Thus, justifying measuring CIMP status at diagnosis. Although clonal selection may occur at relapse, this data supports similar epigenetic characteristics based on CIMP status at diagnosis and relapse.

The observation that our CIMP profile adds prognostic information among relapsed BCP-ALL patients and high-risk (MRD > 0.1% at treatment day 29) T-ALL patients [6] makes our panel a potentially useful prognostic marker for high-risk ALL patients that today lack biological therapy stratifying markers. Therefore, upfront methylation array analysis at diagnosis could be implemented to add epigenetic phenotype information (CIMP status) with potential prognostic relevance, in addition, as a complementary method to support prediction of cytogenetic subtypes [14]. Whether the prognostic relevance of CIMP classification in BCP-ALL could be further improved by including MRD status could not be addressed here since MRD data was not available for the majority of patients, and hence remains to be evaluated in replicative studies of patients treated by contemporary protocols [15].

The biological mechanisms, i.e., genetic aberrations in DNA methylation associated genes or oncogenes remain to be investigated in relation to CIMP status in ALL. Several mechanisms have been shown to influence the epigenetic landscape of ALL cells and recurrent mutations in genes involved in epigenetic regulation have been reported [16, 17]. In a previous analysis of the genes associated with the CpG sites in our CIMP panel, an overrepresentation of transcription factors and polycomb repressive complex target genes was shown [5]. The genes in the CIMP panel were associated with cAMP signaling, but the functional relevance of these associations remains to be shown [5]. Future studies will be needed further evaluate the biology underlying CIMP subgroups.

To conclude, this study together with our recently published study on T-ALL [6] indicates that CIMP classification has the potential to separate high-risk pediatric ALL patients and may confer important information in clinical treatment decision-making.

## Additional file

Additional file 1: Methods description, figures, and tables can be found at the Clinical Epigenetics webpage. (DOCX 282 kb)

## Abbreviations

ALL: Acute lymphoblastic leukemia
BCP-ALL: B-cell precursor acute lymphoblastic leukemia
CIMP: CpG island methylator phenotype
HR/IR/SR: High/intermediate/standard risk
HSCT: Hematopoietic stem cell transplantation
IntReALL: International Study for Treatment of Childhood Relapse ALL
MRD: Minimal residual disease
NOPHO: Nordic Society of Paediatric Haematolgy and Oncology
