# Supplementary material for: DNA methylation holds prognostic information in relapsed precursor B-cell acute lymphoblastic leukemia
Source: Clin Epigenetics. 2018 Mar 5;10:31. doi: 10.1186/s13148-018-0466-3 (PMC5836434; doi:10.1186/s13148-018-0466-3)
Supplement: Supplementary file 1 — Methods description, figures, and tables can be found at the Clinical Epigenetics webpage. (DOCX 282 kb) [file 13148_2018_466_MOESM1_ESM.docx]

**Additional file 1**

**Table S1:** Clinical characteristics of 601 diagnostic pediatric BCP-ALL.

**Table S2:** Primers and amplicon characteristics of the six-gene HRM panel used to verify CIMP classification.

**Table S3: Cox regression analysis.** Cox proportional hazard regression analysis for overall survival in relapse patients.

**Figure S1:** **CIMP classification definition.** The CIMP subgroups were defined based on percentage of methylated CpG sites (methylated CpG site defined as a ß-value ›0,4) within the 1293 CpG site CIMP panel. Since approximately 1/3 of the CpG sites in the CIMP panel seemed to be relevant only for T-ALL the CIMP percentage separating CIMP- and CIMP+ was adjusted for BCP-ALL. Samples with ≤25% methylated CpG sites were classified as CIMP-, and samples > 25% methylated CpG sites were classified as CIMP+.

**Figure S2: Verification of CIMP status.** CIMP classification by methylation array analysis (1293 CpG) was verified by classification based on a six-gene HRM panel selected from the CIMP panel. Mean methylation percentage within the 1293 CpG site methylation array CIMP panel was correlated mean methylation of the six-gene HRM panel.

**Table S1**

| *Diagnostic BCP-ALL patients* | CIMP- n=175 | CIMP+ n=426 | *p-value* |
| --- | --- | --- | --- |
| Sex male/female | 98/77 | 227/199 | *n.s* |
| Age at primary diagnosis (months) | 56 | 60 | *n.s* |
| (median, range) | (12-211) | (12-226) |  |
| WBC x 10^9^/l at primary diagnosis | 14.1 | 12.6 | *n.s* |
| (median, range) | (0.8-986,1) | (0.5-336) |  |
|  |  |  |  |
| Cytogenetics at primary diagnosis |  |  | *<0.001* |
| Favorable ^a^ | 81 (46%) | 241 (57%) |  |
| Unfavorable ^b^ | 36 (21%) | 23 (5%) |  |
| Other ^c^ | 58 (33%) | 162 (38%) |  |
|  |  |  |  |
| Specific cytogenetic aberrations |  |  | *<0.001* |
| dic(9;20) | 2 (10%) | 18 (90%) |  |
| t(1;19) | 19 (83% | 4 (17%) |  |
| t(12;21) | 17 (12%) | 130 (88%) |  |
| t(9;22) | 12 (67%) | 6 (33%) |  |
| <45 | 1 | 3 |  |
| >67 | 0 | 2 |  |
| 11q23 | 4 (29%) | 10 (71%) |  |
| HeH | 64 (37%) | 111 (63%) |  |
| iAMP | 2 | 8 |  |
| Non-recurrent | 23 (25%) | 70 (75%) |  |
| No result | 31 (33%) | 64 (67%) |  |
|  |  |  |  |
| Follow up status |  |  | *n.s* |
| CR1 | 124 | 313 |  |
| induction failure | 2 | 4 |  |
| resistant disease | 3 | 4 |  |
| relapse | 42 | 95 |  |
| DCR1 | 1 | 7 |  |
| SMN | 3 | 3 |  |
|  |  |  |  |
| pCIR_5y_ | 0.20+/-0.02 | 0.21+/-0.03 | *0.520* |
| pEFS_5y_ | 0.76+/-0.02 | 0.75+/-0.03 | *0.424* |
| pOS_5y_ | 0.85+/-0.03 | 0.92+/-0.13 | *0.019* |
| *ns=not significant, WBC=white blood cell count, CR1=first complete remission,* *DCR1=dead in CR1, SMN=second malignancy, pCIR (Cumulative incidence of relapse), pEFS (Event free survival), pOS (Overall survival). ^a^ Favorable: t(12;21)(p12;q22), high hyperdiploidy (modal chromosome number ≥50); ^b^ Unfavorable: t(9;22)(q34;q11),* *t(1;19), MLL rearrangements (11q23), hypodiploidy (modal chromosome number <45); ^c^ Other: non-stratifying or nonspecific cytogenetic aberrations.* | | | |

**Table S2**

| ***Gene Assay*** | ***Primer*** | ***Sequence (5´- 3´)*** | ***Amplicon Size (bp)*** | ***CpGs in amplicon*** |
| --- | --- | --- | --- | --- |
| EYA4  cg26656135 | Forward | GGTGGTYGAAGGGGATGTTTTG | 200 | 17 |
|  | Reverse | TCCCCCRAAAACCCTAAAAC |  |  |
| SMPDL3A  cg14426525 | Forward | TYGTTTGTTGTTTGTTGATTGTTTGG | 200 | 21 |
|  | Reverse | ACCTCTACCRAAAACAACTACCCCCAC |  |  |
| HOXB4  cg08089301 | Forward | TGTGGGTAATTTTTAGAAATTAATGGTTATGAG | 176 | 11 |
|  | Reverse | CCTCCRACTAAAAACTACTCTCTC |  |  |
| FOXA1  cg11260422 | Forward | GTTYGGGTGATTGTAGTTGTTTAG | 197 | 15 |
|  | Reverse | CCTCRCCTTACCTCTCACCTCCTAC |  |  |
| ADAMTS1  cg00472814 | Forward | TTTYGAGTTAGGGGTTATTGTAAAGTTAG | 198 | 14 |
|  | Reverse | AAAACAAACRCAAACAAAATAACTCTAC |  |  |
| ATOH1  cg04107037 | Forward | GTAGAAGAGTGGGTTGAAGTGAAGGAGTTG | 200 | 14 |
|  | Reverse | CAAATACCCTACAAAATAAAAACCAACC |  |  |

**Table S3**

| *Risk factor* | *N* | *Reference group* | *HR* |
| --- | --- | --- | --- |
| CIMP (-/+) | (42/95) | CIMP+ | 1.99 (1.17-3.37)(*p=0.011) |
| IntReALL risk class (relapse site/time to relapse (SR/HR)) | (88/49) | Risk group =SR | 3.27 (1.92-5.54) (*p<0.001) |


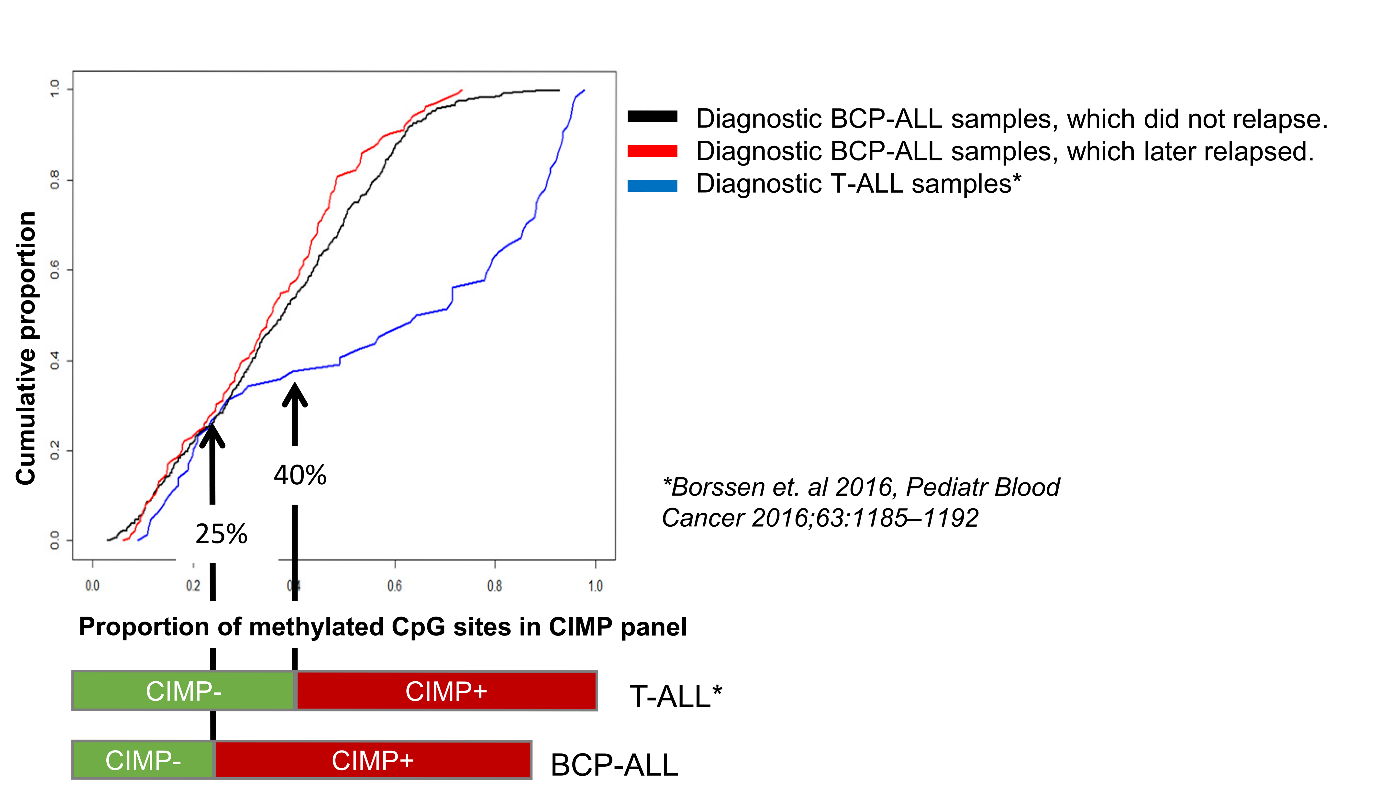
**Figure S1:**


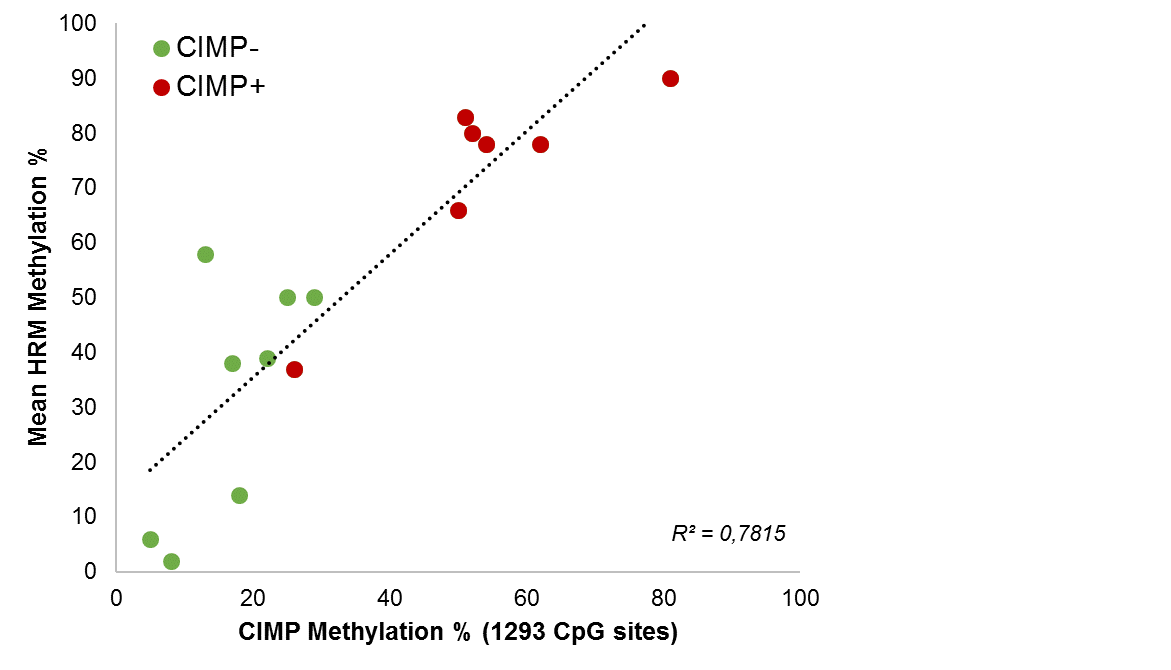
**Figure S2:**

**Materials and methods**

**BCP-ALL samples**

This study included 601 pediatric patients (1-18 years) diagnosed with B-cell precursor ALL (BCP-ALL) between years 1996 and 2008 in the Nordic countries and treated according to the common NOPHO (Nordic society of pediatric hematology and oncology) ALL 1992 and 2000 protocols (Nordlund*, et al* 2013, Schmiegelow*, et al* 2010). Clinical follow up data was extracted from the NOPHO leukemia registry in June 2016 and the mean follow up time for patients was 115 months (range <1-221). The regional and/or national ethics committees approved the study, and the patients and/or their guardians provided informed consent in accordance with the Declaration of Helsinki.

**Methylation array analysis and CpG Island Methylator Phenotype (CIMP) classification**

Infinium HumanMethylation450K BeadChip (Illumina, San Diego, CA, USA) array analysis was previously performed on lymphocytes isolated from bone marrow or peripheral blood cells from ALL patients that included >80% leukemic blasts at diagnosis. The data is publicly available at Gene Expression Omnibus (GEO) with accession number GSE49031 (Nordlund*, et al* 2013). Array data was preprocessed (normalized, filtered for CpG probes that align to multiple genomic locations or within 3 bp from a SNP, and CpG located on X and Y chromosomes were excluded) and samples were classified according to our previously defined CIMP panel (Borssén*, et al* 2016).

The CIMP subgroups were defined based on percentage of methylated CpG sites within the 1293 CpG site CIMP panel (Borssén*, et al* 2016). A specific CpG site was considered methylated if the ß-value was higher than 0,4. Approximately 2/3 of the CpG sites in the CIMP panel, which was originally trained on T-ALL, were relevant for BCP-ALL. Therefore, the CIMP percentage separating CIMP- and CIMP+ was adjusted for the BCP-ALL dataset as samples with ≤25% methylated CpG sites were classified as CIMP-, and samples > 25% methylated CpG sites were classified as CIMP+ (Additional file 1: Figure S1).

**Verification of CIMP classification by HRM analysis**

High resolution melting (HRM) analysis was used to verify the CIMP classification of 15 BCP-ALL patient samples. A six-gene HRM panel was designed based on six CpG sites from the CIMP panel whose methylation profile differentiated the CIMP subgroups. The genes included EYA4, SMPDL3A, HOXB4, FOXA1, ADAMTS1 and ATOH1 (Additional file 1: Table S2). The HRM reaction mix contained 10ng of bisulfite converted DNA (Zymo Research, Irvine, CA), 1X Epitect HRM PCR Mastermix (Qiagen) and 0.75 μM of forward and reverse primers (Table 1). HRM analysis was performed in RotorGene instrument (Qiagen) according to the following program: 95°C for 5 min, followed by 35 cycles of 95°C for 10 sec, 55°C for 30 sec, and 72°C for 20 sec. Melting analysis was conducted by raising the temperature by 0.1° each step from 60 to 90°C. Each assay was run with a standard curve, prepared by diluting 100% methylated DNA (M.SssI treated) in different ratios with DNA from mitogen (wheat germ agglutinin) stimulated primary lymphoblast T-cell cultures P7/R2 (theoretically 0% methylated mononuclear cells). Data was analyzed using Rotor-Gene® software v1.7 (Qiagen).

Mean methylation percentage of the six-gene BCP-ALL HRM panel was calculated and correlated with the methylation percentage of the 1293 CpG site methylation array CIMP panel (Additional file 1: Figure S2). The methylation percentage from the HRM analysis correlated well with the CIMP methylation array analysis (R2 = 0.782, Additional file 1: Figure S2).

**Statistical analysis**

Methylation data analysis was carried out in the R environment (v2.15.0). The Statistical Package for the Social Sciences (SPSS Inc., Chicago, IL) software was used for the statistical analyses. The chi-square and the Mann-Whitney U tests were used to determine whether differences among subgroups existed for discrete and continuous variables, respectively. Estimates of event free survival (pEFS), overall survival (pOS), and cumulative incidence of relapse (pCIR) are given at 5 years and were calculated using the Kaplan-Meier method and subgroups compared using the log rank test. Cox´s proportional hazard regression analysis was used to analyze multiple prognostic factors and estimating hazard ratios (HR) for overall survival with 95% confidence intervals. The significance limit for two-sided p-values was set to <0.05 in all tests. Time in first remission (CR1) was defined as time from diagnosis until first event, comprising induction failure, resistant disease, death in remission, relapse, or second malignant neoplasm. All-cause mortality was the endpoint in the analysis of overall survival (OS) and OS time was either the period from diagnosis to death or relapse to death, and censoring occurred at the date of last known follow up.

Initial risk classification at primary diagnosis in the NOPHO 1992/2000 protocols have been described previously (Taskinen*, et al* 2017), and includes immonophenotype, cytogentic aberrations, WBC count, CNS involvement, and response to induction therapy. Standard/intermediate risk groups were analyzed together since they have similar outcome (Oskarsson*, et al* 2016).

**References**

Borssén, M., Haider, Z., Landfors, M., Noren-Nystrom, U., Schmiegelow, K., Asberg, A.E., Kanerva, J., Madsen, H.O., Marquart, H., Heyman, M., Hultdin, M., Roos, G., Forestier, E. & Degerman, S. (2016) DNA Methylation Adds Prognostic Value to Minimal Residual Disease Status in Pediatric T-Cell Acute Lymphoblastic Leukemia. *Pediatr Blood Cancer,* **63,** 1185-1192.

Nordlund, J., Backlin, C.L., Wahlberg, P., Busche, S., Berglund, E.C., Eloranta, M.L., Flaegstad, T., Forestier, E., Frost, B.M., Harila-Saari, A., Heyman, M., Jonsson, O.G., Larsson, R., Palle, J., Ronnblom, L., Schmiegelow, K., Sinnett, D., Soderhall, S., Pastinen, T., Gustafsson, M.G., Lonnerholm, G. & Syvanen, A.C. (2013) Genome-wide signatures of differential DNA methylation in pediatric acute lymphoblastic leukemia. *Genome Biol,* **14,** r105.

Oskarsson, T., Soderhall, S., Arvidson, J., Forestier, E., Montgomery, S., Bottai, M., Lausen, B., Carlsen, N., Hellebostad, M., Lahteenmaki, P., Saarinen-Pihkala, U.M., Jonsson, O.G., Heyman, M., Nordic Society of Paediatric, H. & Oncology, A.L.L.r.w.g. (2016) Relapsed childhood acute lymphoblastic leukemia in the Nordic countries: prognostic factors, treatment and outcome. *Haematologica,* **101,** 68-76.

Schmiegelow, K., Forestier, E., Hellebostad, M., Heyman, M., Kristinsson, J., Soderhall, S. & Taskinen, M. (2010) Long-term results of NOPHO ALL-92 and ALL-2000 studies of childhood acute lymphoblastic leukemia. *Leukemia,* **24,** 345-354.

Taskinen, M., Oskarsson, T., Levinsen, M., Bottai, M., Hellebostad, M., Jonsson, O.G., Lahteenmaki, P., Schmiegelow, K. & Heyman, M. (2017) The effect of central nervous system involvement and irradiation in childhood acute lymphoblastic leukemia: Lessons from the NOPHO ALL-92 and ALL-2000 protocols. *Pediatr Blood Cancer,* **64,** 242-249.
